# Targeting glycolytic reprogramming by tsRNA-0032 for treating pathological lymphangiogenesis

**DOI:** 10.1038/s41419-025-07366-w

**Published:** 2025-01-28

**Authors:** Fan Ye, Ziran Zhang, Lianjun Shi, Shuting Lu, Xiumiao Li, Wan Mu, Qin Jiang, Biao Yan

**Affiliations:** 1 The Affiliated Eye Hospital, Nanjing Medical University, Nanjing, China; 2 Department of Ophthalmology, Shanghai General Hospital, Shanghai Jiao Tong University School of Medicine, Shanghai, China; 3 Eye Institute and Department of Ophthalmology, Eye & ENT Hospital, Fudan University, Shanghai, China

**Keywords:** Genetics research, Endocrine system and metabolic diseases

## Abstract

Lymphangiogenesis is vital for tissue fluid homeostasis, immune function, and lipid absorption. Abnormal lymphangiogenesis has been implicated in several diseases such as cancers, inflammatory, and autoimmune diseases. In this study, we elucidate the role of tsRNA-0032 in lymphangiogenesis and its molecular mechanism. tsRNA-0032 expression is significantly decreased in corneal suture model and human lymphatic endothelial cell (HLEC) model under inflammatory condition. Overexpression of tsRNA-0032 exerts anti-lymphangiogenic effects by inhibiting HLEC proliferation, migration, and tube formation. Moreover, overexpression of tsRNA-0032 inhibits suture-induced corneal lymphangiogenesis. tsRNA-0032 is mainly located in the cytoplasm and interacts with Ago2 protein. Overexpression of tsRNA-0032 reduces ATP production and decreases pyruvate and lactate levels by targeting PKM2, a key enzyme in glycolysis. This regulation of glycolysis alters cellular energy and metabolic balance in HLECs, contributing to anti-lymphangiogenic effects. Clinical data reveals that tsRNA-0032 levels are significantly reduced in corneal tissues of transplant recipients compared to donors, while PKM2 expression is elevated, highlighting the clinical relevance of tsRNA-0032/PKM2 axis in corneal lymphangiogenesis. This study offers new insights into the regulation of lymphangiogenesis and presents potential therapeutic targets for lymphangiogenesis-related diseases.

## Introduction

The lymphatic vessel system is an important component of circulatory system, working in concert with vascular system to maintain fluid balance, support immune function, and facilitate lipid absorption [1]. Lymphangiogenesis, the process of generating new lymphatic vessels, is primarily controlled by lymphatic endothelial cells (LECs). LECs typically maintain a quiescent state except during embryonic development [2]. Under pathological conditions such as tumor metastasis [3], cardiovascular diseases [4], and various inflammatory diseases, LECs are activated, stimulating the proliferation, migration, and subsequent formation of new lymphatic vasculature. Within ocular environment, abnormal lymphatic vessels can contribute to keratitis, dry eye, and transplantation rejection [5–7].

Although significant research has been conducted, the understanding of lymphangiogenesis is still insufficient. As a result, clinical approaches targeting lymphatic vessels remain highly restricted. Current therapeutic strategies predominantly focus on inhibiting VEGFR3 or targeting LEC-autonomous activation through rapamycin, gene therapy, or surgical resection [8, 9]. Rapamycin, an mTOR inhibitor, has long been a pivotal component in organ transplantation, owing largely to its powerful ability to suppress immune responses [10]. While lymphangiogenesis holds promise as a therapeutic target for the conditions involving inflammation and immune system diseases, its long-term effectiveness and potential side effects remain uncertain. The complex nature of lymphatic biology necessitates a carefully crafted approach when considering this strategy. A comprehensive understanding of lymphangiogenesis is crucial for developing innovative therapies to address diseases characterized by pathological lymphangiogenesis.

The occurrence and progression of pathological lymphangiogenesis require a complex interplay of various cytokines and growth factors. Existing studies have shown that small extracellular vesicles (sEVs), pro-inflammatory cytokines, ROS, and various non-coding RNAs can regulate lymphangiogenesis [11–13]. sEVs play a crucial role in the transport and distribution within lymphatic system. When sEVs are injected subcutaneously into the tail base and tumor tissues, they preferentially accumulate in lymph nodes. Pro-inflammatory cytokines and ROS promote lymphangiogenesis by regulating the proliferation and migration of LECs.

Non-coding RNAs also can regulate the function of LECs and lymphangiogenesis by regulating gene expression. tRNA-derived small RNAs (tsRNAs) have emerged as a novel class of small non-coding RNAs, arising from the cleavage of precursor or mature tRNAs. These cleavage events are predominantly triggered by stress, such as amino acid deficiency, UV radiation, hypoxia, oxidative damage, and viral infection. The production of tsRNAs is limited under normal condition, highlighting their role as tRNA-derived stress-induced RNAs, suggesting that tsRNAs may play a pivotal role in stress response, potentially regulating gene expression and maintaining cellular homeostasis [14]. Emerging evidence underscores the capacity of tsRNAs to modify RNAs and interact with proteins, thereby exerting substantial influence over a range of biological processes, including gene silencing, ribosome biogenesis, and epigenetic regulation [15]. Increasing studies have also revealed the involvement of tsRNAs in pathological processes, such as tumor metastasis, systemic inflammation, and lipid absorption disorders [16–18]. Given the established link between tsRNAs and angiogenesis [19, 20], it is also imperative to investigate their potential roles in lymphangiogenesis.

In this study, we investigated the role of tsRNA-0032, a specific cleavage product of tRNA-GTG-His, in lymphangiogenesis. Our findings demonstrate that tsRNA-0032 expression is down-regulated during lymphangiogenesis, while its up-regulation attenuates lymphangiogenesis and suppresses LEC hyperactivation under inflammatory conditions. Mechanistically, tsRNA-0032 regulates lymphangiogenesis by modulating glycolysis. These findings position tsRNA-0032 as a potential regulator of LEC dysfunction.

## Materials and Methods

### Ethics approval and consent to participate

All animals were obtained from Nanjing Junke Bioengineering Corporation and treated in accordance with the Association for Research in Vision and Ophthalmology’s guidelines for the use of animals in ophthalmic and vision research. All procedures were approved by the Animal Experiment Management Committee of the author’s institute (Approval number: 20221018). Corneal tissue samples were collected from keratitis patients who received corneal transplantation. The clinical studies were conducted according to the Declaration of Helsinki and approved by the Ethics Committee of Eye Hospital. Informed consent was obtained from all participants.

### Corneal suture model

Corneal lymphangiogenesis model was established to induce inflammation and stimulate lymphatic vessel growth towards corneal center. Male C57BL/6 J mice (6-8 weeks old) were anesthetized with an intraperitoneal injection of xylazine and ketamine mixture. Pupil dilation was achieved using phenylephrine and tropicamide eye drops. The interrupted 10-0 nylon sutures were placed in corneal stroma near the limbus, spaced 120 degrees apart. To prevent infection, gatifloxacin ointment was applied topically. Corneas were harvested seven days post-surgery for subsequent analysis.

### Matrigel plug assay

Male C57BL/6 J mice (6–8 weeks old) were subcutaneously injected with 500 µL of Matrigel (ABW, China, 00827235) supplemented with heparin (60 units) and VEGF (100 ng/mL, PEPROTECH). Each Matrigel mixture contained either negative control (NC) agomir, tsRNA-0032 agomir, NC antagomir, or tsRNA-0032 antagomir. After 14 days, the Matrigel plugs were harvested, cryosectioned, and stained with LYVE-1 antibody (1:200, Abcam, USA, ab14917), followed by an Alexa Fluor 594 secondary antibody (1:500, Invitrogen, USA, 2506100).

### Cell culture and transfection

HLECs were cultured in Endothelial Cell Basal Medium MV (Promocell, Germany, C-22220) supplemented with 12% fetal bovine serum (ScienCell, USA) and penicillin/streptomycin (100 U/mL/100 µg/mL; Gibco, USA) in a humidified incubator at 37 °C with 5% CO_2_. tsRNA-0032 mimics, inhibitors, and their respective negative controls were transfected into HLECs using Lipofectamine 3000 (Invitrogen, USA, L3000015) according to the manufacturer’s protocol when cells reached approximately 80% confluence.

### Fluorescence in situ hybridization (FISH)

The cellular localization of tsRNA-0032 in HLECs was determined by FISH assay. Cy3-labeled probes, 5’-CGAACCGAGGTTGCTGCGGCC-3’, specific to tsRNA-0032 were designed and synthesized by Servicebio (Wuhan, China). The signals of the probes were detected by a Fluorescent In Situ Hybridization Kit according to the manufacturer’s instruction.

### Nucleoplasmic separation assay

Nuclear and cytoplasmic RNAs from HLECs were isolated using the Cytoplasmic and Nuclear RNA Purification Kit following the manufacturer’s instruction. The extracted RNAs were transcribed into cDNAs and analyzed via qRT-PCR assays. The 2^−ΔΔCt^ method was used to determine relative gene expression in the nuclear and cytoplasmic fractions.

### RNA immunoprecipitation (RIP)

RIP was performed using the Magna RIP RNA-Binding Protein Immunoprecipitation Kit (Millipore, USA, 17-701) according to the manufacturer’s protocol. Antibodies targeting Ago2, PKM2, or IgG were incubated with the magnetic beads overnight at 4 °C. Following immunoprecipitation, the co-precipitated RNAs were extracted and subjected to qRT-PCR assay to detect the levels of tsRNA-0032 and PKM2 expression.

### Statistical analysis

Student’s *t*-test was employed to compare differences between two groups, while one-way analysis of variance (ANOVA) was used for comparison among multiple groups. The normality of data distribution was confirmed prior to ANOVA. The results were shown as mean ± standard deviation, with statistical significance defined as *P* < 0.05. All analyses were conducted using GraphPad Prism 8 software (GraphPad Software, USA).

## Results

### tsRNA-0032 expression is down-regulated during inflammatory stress

**Fig. 1:**
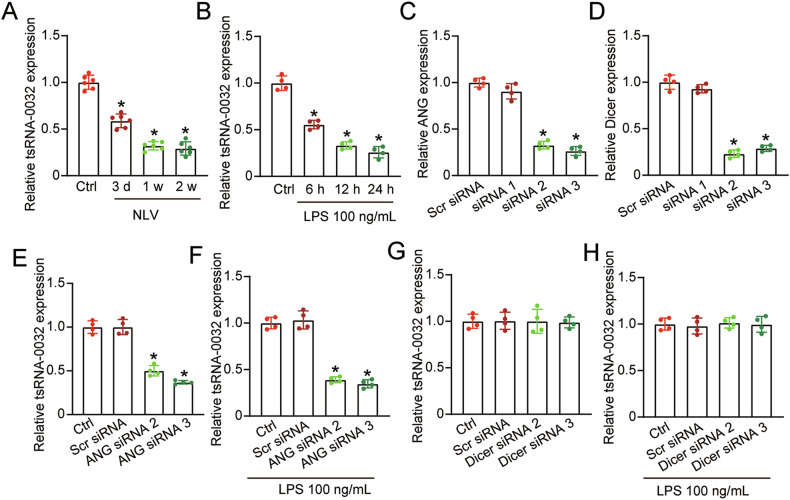
tsRNA-0032 expression is down-regulated during inflammatory stress. **A** qRT-PCR assays were conducted to evaluate the expression levels of tsRNA-0032 in the corneas of C57BL/6 mice from the untreated control group (Ctrl) and the suture groups at 3 days, 1 week, and 2 weeks (*n* = 6). **B** qRT-PCR assays were conducted to compare tsRNA-0032 expression levels in HLECs exposed to inflammatory stress (LPS, 100 ng/mL) for 6, 12, and 24 h, or left untreated (Ctrl) (*n* = 4). **C, D** HLECs were transfected with ANG siRNA 1-3, Dicer siRNA 1-3, scramble (Scr) siRNA as a negative control, or left untreated (Ctrl) for 24 h. qRT-PCR assays were conducted to detect ANG and Dicer expression levels (*n* = 4). **E**–**H** HLECs were transfected with ANG siRNA2/3, Dicer siRNA2/3, Scr siRNA as a negative control, or left untreated (Ctrl), and incubated with or without LPS (100 ng/mL) for 24 h. qRT-PCR assays were conducted to detect tsRNA-0032 expression levels (*n* = 4). One-way ANOVA followed by a post hoc Bonferroni test; *P < 0.05 versus Ctrl group.

Pathological conditions such as inflammation or trauma can induce lymphatic vessel growth into the corneal center [21]. To investigate the relationship between tsRNA-0032 expression and lymphangiogenesis, we employed a corneal suture model in vivo and lipopolysaccharide (LPS)-induced lymphangiogenesis model in vitro. In the corneal suture mouse model, qRT-PCR analysis demonstrated a significant down-regulation of tsRNA-0032 expression in sutured corneas compared to the controls (Fig. 1A). LPS-induced HLECs exhibited decreased tsRNA-0032 levels (Fig. 1B). Given that tsRNAs are generated from tRNAs by ANG and Dicer [22], we explored the underlying mechanism of tsRNA-0032 down-regulation. We silenced ANG or Dicer in HLECs under normal condition and LPS-treated condition. The results showed that transfection of ANG siRNA 2/3 or Dicer siRNA 2/3 led to reduced levels of ANG or Dicer expression (Fig. 1C, D). ANG knockdown significantly reduced tsRNA-0032 expression, while Dicer silencing had no effect on tsRNA-0032 expression both under normal condition and LPS-treated condition (Fig. 1E–H). These results suggest that tsRNA-0032 acts as a potential regulator of lymphangiogenesis, and its downregulation in pathological conditions is primarily mediated by ANG.

### tsRNA-0032 inhibits lymphatic endothelial cell function in vitro

**Fig. 2:**
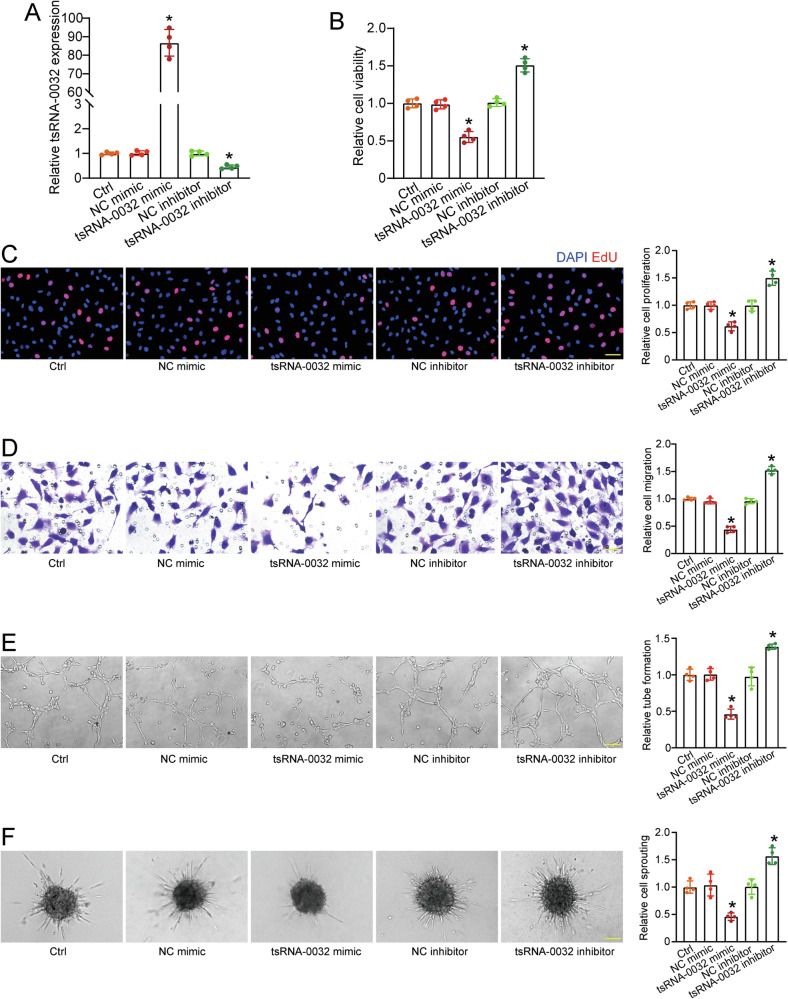
tsRNA-0032 inhibits lymphatic endothelial cell function in vitro. **A**–**F** HLECs were transfected with negative control (NC) mimics, tsRNA-0032 mimics, NC inhibitors, tsRNA-0032 inhibitors, or left untreated (Ctrl) for 6 h and then exposed to LPS (100 ng/mL) for 12 h. The expression levels of tsRNA-0032 were determined by qRT-PCRs (**A**, *n* = 4). Cell viability was detected by CCK-8 assay (**B**, *n* = 4). Cell proliferation was detected by EdU staining. EdU, red; DAPI, blue. Scale bar, 20 μm (**C**, *n* = 4). Cell migration was detected by transwell assays. Scale bar, 20 μm (**D**, *n* = 4). Tube formation ability was detected by Matrigel assays. Scale bar, 100 μm (**E**, *n* = 4). Cell sprouting ability was detected by spheroid sprouting assays. Scale bar, 100 μm (**F**, *n* = 4). One-way ANOVA followed by Bonferroni’s post hoc test; ^*^P < 0.05 versus Ctrl group.

To investigate the role of tsRNA-0032 in lymphangiogenesis, HLECs were transfected with tsRNA-0032 mimics or inhibitors. Compared to the control group, transfection with tsRNA-0032 mimics significantly increased tsRNA-0032 expression, while transfection with tsRNA-0032 inhibitors led to a marked reduction of tsRNA-0032 expression (Fig. 2A). Cell viability was assessed using CCK-8 assays, which demonstrated that overexpression of tsRNA-0032 reduced cell viability, while silencing of tsRNA-0032 increased it (Fig. 2B). 5-Ethynyl-2’-deoxyuridine (EdU) assays demonstrated that overexpression of tsRNA-0032 decreased HLEC proliferation, while silencing of tsRNA-0032 promoted proliferation (Fig. 2C). Transwell assays revealed that overexpression of tsRNA-0032 significantly inhibited HLEC migration, whereas silencing of tsRNA-0032 led to increased migration (Fig. 2D). Transfection with tsRNA-0032 mimics reduced the formation of tube-like structures and sprouting, while the inhibitors enhanced these processes (Fig. 2E, F). These results indicate that tsRNA-0032 regulates the biological functions of HLECs in vitro.

### tsRNA-0032 inhibits pathological lymphangiogenesis in vivo

**Fig. 3:**
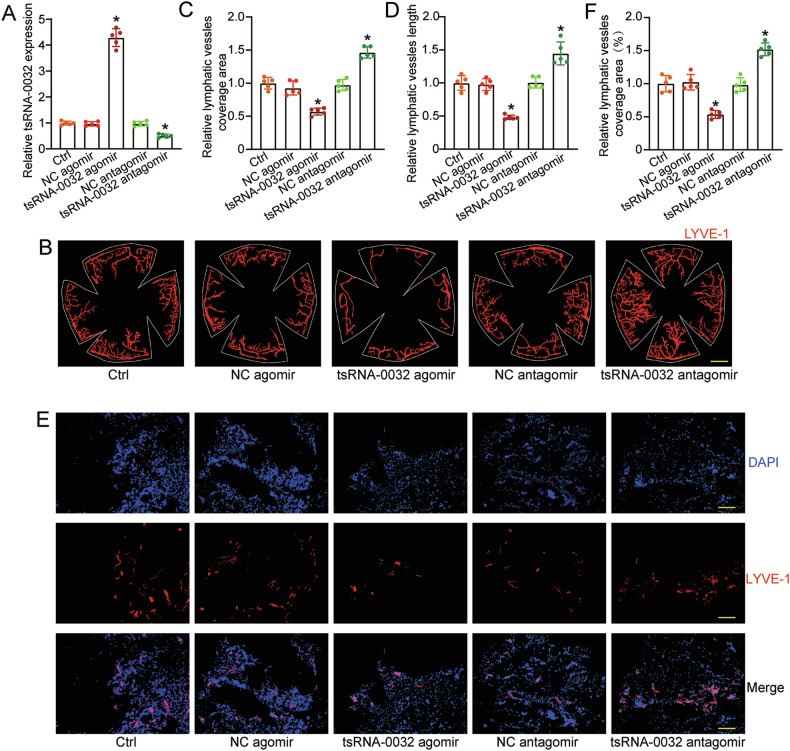
tsRNA-0032 inhibits pathological lymphangiogenesis in vivo. **A** C57BL/6 mice were transfected with negative control (NC) agomir, tsRNA-0032 agomir, NC antagomir, or tsRNA-0032 antagomir using subconjunctival injection, or left untreated (Ctrl). qRT-PCRs were conducted to detect tsRNA-0032 expression levels (*n* = 5, *P < 0.05, one-way ANOVA followed by post hoc Bonferroni test). **B**–**D** C57BL/6 mice underwent corneal suture placement, which remained in place for 7 days. Neolymphatic vessels in the flat-mounted cornea were visualized using the fluorescence signal of LYVE-1 (*n* = 5). Scale bar: 200 μm. Quantification of neolymphatic vessel coverage area (**C**) and total length (**D**) was conducted (*n* = 5). **E**, **F** C57BL/6 mice received subcutaneous injections with Matrigel, mixed with NC agomir, tsRNA-0032 agomir, NC antagomir, tsRNA-0032 antagomir, or left untreated (Ctrl). The matrigel plugs were harvested at day 14 after injection. The frozen sections were stained with DAPI (blue) and LYVE-1 antibody (red) (*n* = 5). Quantification of neolymphatic vessel coverage area was conducted (*n* = 5, *P < 0.05, one-way ANOVA followed by post hoc Bonferroni test, *P < 0.05 versus Ctrl group).

To investigate the role of tsRNA-0032 in lymphangiogenesis in vivo, a suture-induced corneal lymphangiogenesis model was employed. tsRNA-0032 agomir or antagomir was injected into the conjunctival sac to regulate tsRNA-0032 expression, as confirmed by qRT-PCRs (Fig. 3A). Up-regulation of tsRNA-0032 via agomir injection reduced corneal lymphangiogenesis, while down-regulation via antagomir injection increased it, as assessed by LYVE-1 immunofluorescence staining (Fig. 3B–D).

The Matrigel plug assay was conducted to further elucidate the role of tsRNA-0032 in lymphangiogenesis in vivo. Matrigels were mixed with either negative control (NC) agomir, tsRNA-0032 agomir, NC antagomir, or tsRNA-0032 antagomir, and then injected subcutaneously into the groin regions of the mice. To facilitate the growth of neo-lymphatic vessels, VEGF-C was added to each Matrigel prior of injection. After a period of 14 days, the Matrigels were harvested for subsequent analysis. Immunofluorescence staining was performed using the specific antibody against LYVE-1. The results showed that tsRNA-0032 up-regulation led to a marked reduction of lymphangiogenesis, whereas tsRNA-0032 downregulation resulted in an increase in lymphangiogenesis (Fig. 3E, F). These results suggest that tsRNA-0032 is involved in the process of pathological lymphangiogenesis.

### tsRNA-0032 regulates HLEC function via targeting PKM2

**Fig. 4:**
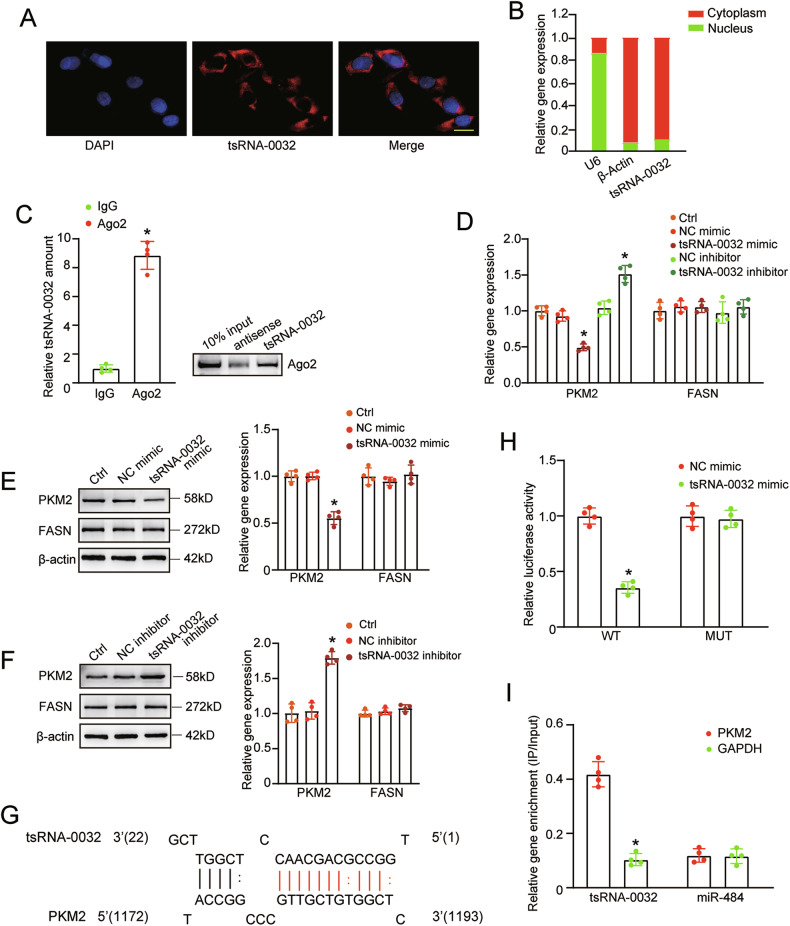
tsRNA-0032 regulates HLECs function by targeting PKM2. **A** Fluorescence in situ hybridization (FISH) assays were conducted to detect the intracellular distribution of tsRNA-0032. Scale bar: 10 μm. **B** Nucleoplasmic separation assays were conducted to quantify the intracellular distribution of tsRNA-0032, with β-Actin and U6 serving as the cytoplasmic and nuclear controls, respectively. **C** Cellular fractions were isolated from HLECs and immunoprecipitated using Ago2 or IgG antibodies. The amount of tsRNA-0032 in the immunoprecipitates was determined by qRT-PCRs (*n* = 4, *P < 0.05, Student’s *t*-test). Western blots were conducted to detect the specific interaction between tsRNA-0032 and Ago2 (*n* = 4). **D** qRT-PCR assays were conducted to detect mRNA expression levels of PKM2 and FASN in HLECs transfected with negative control (NC) mimics, tsRNA-0032 mimics, NC inhibitors, tsRNA-0032 inhibitors, or left untreated (Ctrl) (*n* = 4, *P < 0.05, one-way ANOVA followed by Bonferroni test). **E**, **F** Western blots were conducted to detect the levels of PKM2 and FASN protein in HLECs transfected with NC mimics, tsRNA-0032 mimics, NC inhibitors, tsRNA-0032 inhibitors, or left untreated (Ctrl) (*n* = 4, one-way ANOVA followed by Bonferroni’s post hoc test, *P < 0.05 versus Ctrl group). **G** Nucleobase complementary alignment between tsRNA-0032 and PKM2 is shown. **H** The luciferase activity of WT-Luc-PKM2 or mutant Luc-PKM2 was determined after transfection with tsRNA-0032 mimics or NC mimics in HLECs (*n* = 4, Student’s *t*-test, *P < 0.05 versus Ctrl group). **I** The 3ʹ-end biotinylated tsRNA-0032 or biotinylated miR-484 was transfected into HLECs. After streptavidin capture, the levels of PKM2 and GAPDH in the input and bound fractions were detected by qRT-PCRs (*n* = 4, Student’s *t*-test, *P < 0.05 versus Ctrl group).

To elucidate the mechanism underlying tsRNA-0032-mediated lymphangiogenesis, we first investigated the subcellular localization of tsRNA-0032 in HLECs using fluorescence in situ hybridization (FISH). The results revealed that tsRNA-0032 was predominantly localized to the cytoplasm (Fig. 4A). Nucleo-cytoplasmic fractionation assays further confirmed the predominantly cytoplasmic localization of tsRNA-0032 (Fig. 4B). Given that Ago2 is a key cytoplasmic protein involved in post-transcriptional gene regulation mediated by tsRNAs [23, 24], we performed RIP assays to investigate whether tsRNA-0032 exerted its function through binding to Ago2. RIP assays revealed that Ago2 immunoprecipitated significantly more tsRNA-0032 compared to the negative control IgG (Fig. 4C).

Using the tRFTar database (http://www.rnanut.net/tRFTar/), we next analyzed the potential downstream pathways and target genes of the tsRNA-0032/Ago2 complex. The database predicted that tsRNA-0032 regulated cellular glycolysis by targeting PKM2 and altered fatty acid synthesis by targeting fatty acid synthase (FASN). To further verify the target gene of tsRNA-0032 in HLECs, qRT-PCRs and western blots were performed. tsRNA-0032 overexpression significantly decreased the levels of PKM2 mRNA and protein expression, while its silencing exhibited the opposite effects. In contrast, the levels of FASN mRNA and protein remained unaffected following tsRNA-0032 modulation (Fig. 4D–F).

Base pairing complementarity between tsRNA-0032 and PKM2 was confirmed (Fig. 4G). Luciferase reporter activity assays demonstrated that tsRNA-0032 overexpression significantly reduced the luciferase activity of wild-type PKM2 3’-UTR, but not its mutant form, indicating the direct interaction between tsRNA-0032 and PKM2 3’-UTR (Fig. 4H). RNA pull-down assays revealed that PKM2 was specifically enriched in tsRNA-0032-bound fractions compared to miR-484-bound fractions (Fig. 4I). Collectively, these results suggest that tsRNA-0032 regulates HLEC function by targeting PKM2 via binding to Ago2 protein.

### tsRNA-0032/PKM2 signaling axis alters glycolysis in HLECs

**Fig. 5:**
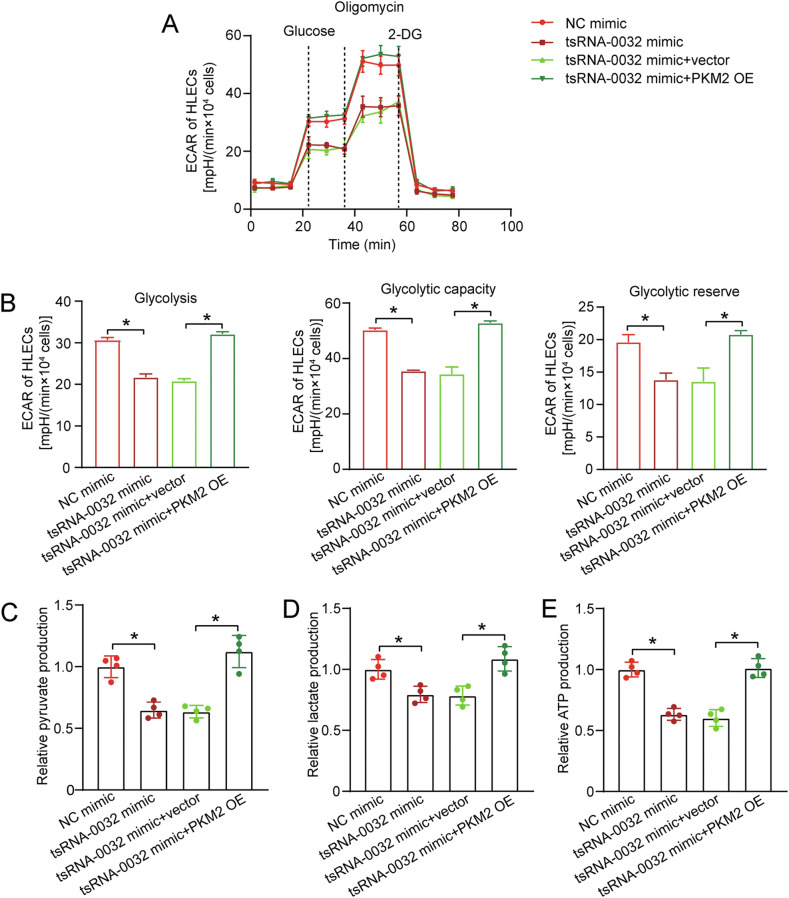
tsRNA-0032/PKM2 signaling axis alters glycolysis in HLECs. **A**–**E** HLECs were transfected with negative control (NC) mimics, tsRNA-0032 mimics, tsRNA-0032 plus null vector, or tsRNA-0032 plus PKM2 overexpression vector. The Seahorse XFe96 Analyzer was used to detect the extracellular acidification rate (ECAR) (**A**). Glycolysis, glycolytic capacity, and glycolytic reserve were calculated from ECAR results (**B**). Pyruvate, lactate, and ATP production in HLECs were detected using pyruvate quantification, lactate quantification, and ATP quantification kits, respectively (**C**–**E**, *n* = 4). One-way ANOVA followed by the Bonferroni test; *P < 0.05 between the marked groups.

PKM2 is the rate-limiting enzyme in the final step of glycolysis, catalyzing the conversion of phosphoenolpyruvate to pyruvate [25]. tsRNA-0032/PKM2 signaling axis regulates lymphatic endothelial cell function by modulating cellular glycolysis. To assess glycolytic activity, extracellular acidification rate (ECAR) was measured in HLECs using a Seahorse XF extracellular flux analyzer. tsRNA-0032 overexpression suppressed glycolysis, glycolytic capacity, and glycolytic reserve, while PKM2 overexpression partially rescued the inhibitory effect (Fig. 5A, B). Conversely, tsRNA-0032 knockdown enhanced glycolytic activity, which was attenuated by PKM2 silencing (Fig. S1A, B).

To further assess the effects of altered tsRNA-0032 expression on glycolysis, pyruvate and lactate production were quantified. Overexpression of tsRNA-0032 resulted in decreased levels of pyruvate and lactate, with these effects being partially reversed by PKM2 overexpression (Fig. 5C, D). Conversely, knockdown of tsRNA-0032 led to increased pyruvate and lactate production, an effect that was reversed by PKM2 silencing (Fig. S1C, D). Given the critical role of glycolysis in ATP production, ATP levels were measured. Overexpression of tsRNA-0032 reduced ATP production, which was rescued by PKM2 overexpression, whereas knockdown of tsRNA-0032 increased ATP production, an effect that was attenuated by PKM2 silencing (Fig. 5E, Fig. S1E). To determine the specificity of tsRNA-0032’s effects on glycolysis, the expression levels of other key glycolytic enzymes, HK2 and PFKFB3, were measured, and no significant changes were found (Fig. S1F). Collectively, these results indicate that tsRNA-0032/PKM2 signaling axis specifically regulates glycolysis in HLECs.

### tsRNA-0032/PKM2 signaling axis is involved in lymphangiogenesis in vitro

To verify whether the tsRNA-0032/PKM2 signaling axis is involved in lymphangiogenesis, we examined whether PKM2 alone could influence lymphangiogenesis in vitro. The expression of PKM2 was modulated using a PKM2 pcDNA3.1 vector for overexpression and PKM2 siRNA for silencing, with the efficiency of these interventions confirmed by qRT-PCRs and western blots (Fig. S2A, B). Overexpression of PKM2 enhanced HLEC proliferation, migration, tube formation, and sprouting activity, whereas silencing PKM2 impaired these cellular functions (Fig. S2C–F).

**Fig. 6:**
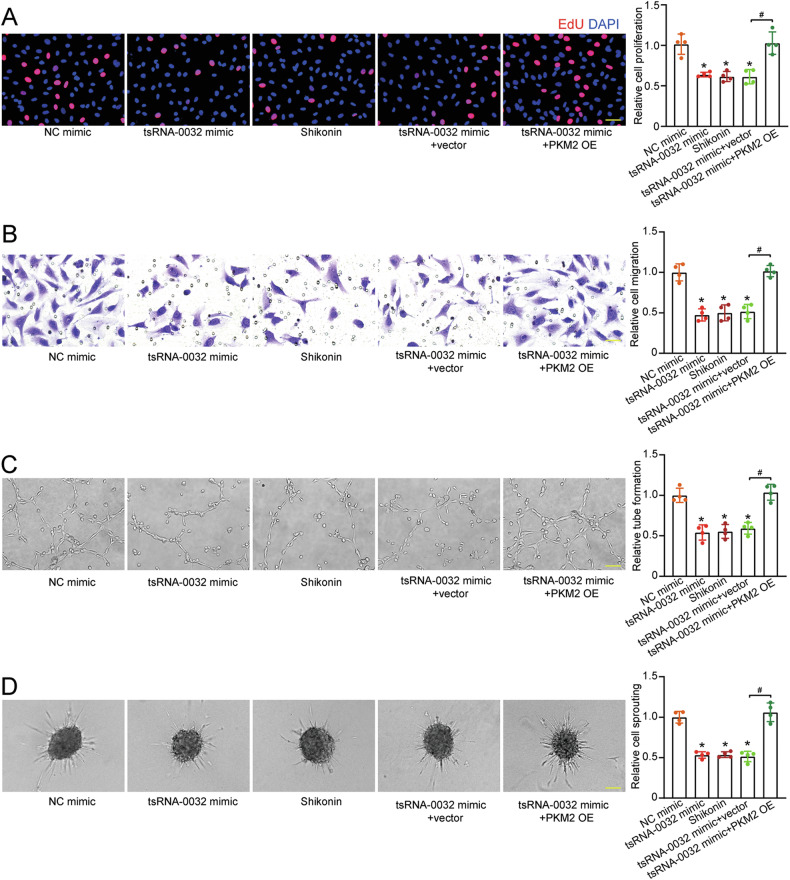
tsRNA-0032/PKM2 signaling axis is involved in lymphangiogenesis in vitro. HLECs were transfected with negative control (NC) mimics, tsRNA-0032 mimics, tsRNA-0032 plus null vector, tsRNA-0032 plus PKM2 overexpression vector, or treated with shikonin for 6 h, followed by exposure to LPS (100 ng/mL) for 12 h. Cell proliferation was detected by EdU staining. EdU, red; DAPI, blue. Scale bar, 20 μm (**A**, *n* = 4). Cell migration was detected by Transwell assays. Scale bar, 20 μm (**B**, *n* = 4). Tube formation ability was detected by Matrigel assays. Scale bar, 100 μm (**C**, *n* = 4). Sprouting ability was detected by spheroid sprouting assays. Scale bar, 100 μm (**D**, *n* = 4). One-way ANOVA followed by Bonferroni test; ^*^P < 0.05 versus NC mimic group, ^#^*P* < 0.05 between the marked groups.

To investigate whether modulating PKM2 activity could rescue lymphatic dysfunction induced by tsRNA-0032, we used shikonin, a specific inhibitor of PKM2 activity with no reported effects on PKM1 [26, 27]. CCK-8 assays demonstrated that the concentrations of shikonin below 1 μM were non-toxic to HLECs (Fig. S3). To further assess the functional rescue of tsRNA-0032-mediated HLEC dysfunction, PKM2 was overexpressed. PKM2 overexpression effectively reversed the inhibitory effects of tsRNA-0032 on HLEC proliferation, migration, tube formation, and sprouting (Fig. 6A–D). Collectively, these findings demonstrate that tsRNA-0032/PKM2 signaling axis plays a critical role in regulating lymphatic endothelial function.

### tsRNA-0032/PKM2 signaling axis is involved in regulating lymphangiogenesis in vivo

**Fig. 7:**
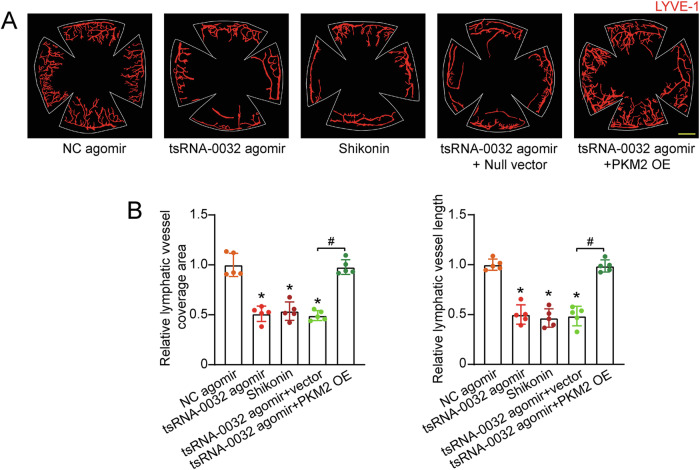
tsRNA-0032/PKM2 signaling axis is involved in lymphangiogenesis in vivo. **A** C57BL/6 mice were administered negative control (NC) agomir, tsRNA-0032 agomir, tsRNA-0032 combined with PKM2 null vector, tsRNA-0032 combined with PKM2 overexpression vector, or treated with shikonin, followed by corneal suture placement. Neolymphatic vessels in the flat-mounted corneas were visualized using the fluorescence signal of LYVE-1. Scale bar: 200 μm. **B** Quantification of neolymphatic vessel coverage area and total length was performed. *n* = 5; One-way ANOVA followed by post hoc Bonferroni test; *P < 0.05 versus Ctrl group.

To elucidate the in vivo functional significance of tsRNA-0032/PKM2 signaling axis in lymphangiogenesis, a corneal suture model was established. Injection of tsRNA-0032 agomir into the conjunctival sac led to a significant reduction in the corneal lymphangiogenic area, an effect that was similarly observed following treatment with shikonin, a specific PKM2 inhibitor. Conversely, overexpression of PKM2 through vector-mediated gene delivery effectively counteracted the anti-lymphangiogenic effects induced by tsRNA-0032 agomir, as shown by increased formation and length of new lymphatic vessels (Fig. 7A, B). Collectively, these results underscore the critical role of tsRNA-0032/PKM2 signaling axis in corneal lymphangiogenesis.

### Clinical relevance of tsRNA-0032-mediated signaling in corneal lymphangiogenesis

**Fig. 8:**
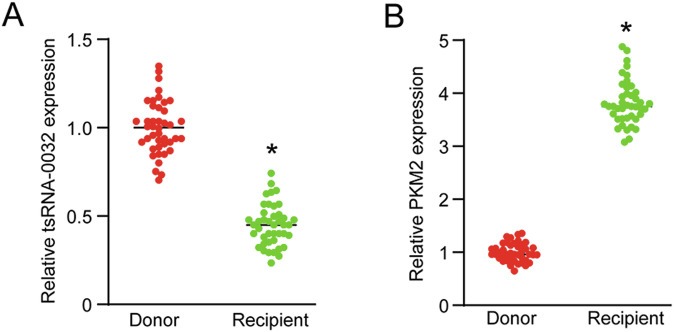
Clinical relevance of tsRNA-0032-mediated signaling in corneal lymphangiogenesis. **A** qRT-PCR assays were conducted to detect the levels of tsRNA-0032 expression in donors and recipients of corneal allograft transplantation (*n* = 42). **B** qRT-PCR assays were conducted to detect the levels of PKM expression in donors and recipients of corneal allograft transplantation (*n* = 42). Student’s *t*-test. *P < 0.05 versus the donor group.

Keratitis, an inflammation of the cornea, is often associated with corneal lymphangiogenesis [28, 29]. To investigate the potential clinical implications of tsRNA-0032/PKM2 signaling axis, corneal tissue samples were collected from keratitis patients who received corneal transplantation (recipients) and healthy individuals (donors). qRT-PCR analysis revealed a significant down-regulation of tsRNA-0032 and a concomitant up-regulation of PKM2 in the corneal tissue of keratitis patients compared to healthy controls (Fig. 8A, B). These findings reveal a correlation between the dysregulation of tsRNA-0032/PKM2 signaling axis and corneal lymphangiogenesis in keratitis patients. Additionally, we compared the expression levels of tsRNA-0032 and PKM2 among recipients with different types of keratitis. qRT-PCR assays indicated no significant differences in the expression of tsRNA-0032 and PKM2 among patients with viral keratitis, bacterial keratitis, fungal keratitis, and immune keratitis (Fig. S4).

## Discussion

Although physiological lymphangiogenesis is rare in healthy adults, pathological lymphangiogenesis plays a significant role in various disease processes, including inflammation, lymphedema, organ transplant rejection, tumor metastasis, and cardiovascular disease [30]. Under inflammatory conditions such as rheumatoid arthritis, inflammation-induced lymphangiogenesis regulates fluid drainage, immune cell migration, and the clearance of inflammatory mediators, ultimately aiding in the resolution of inflammation [31]. Conversely, following organ transplantation, lymphangiogenesis can trigger immune system reactivation in draining lymph nodes, potentially leading to organ rejection [32]. In cancer, tumor cells spread to lymph nodes via lymphatic system, and inhibiting tumor lymphangiogenesis and lymph node metastasis in animal models effectively impedes tumor progression [33, 34]. A common feature among these lymphangiogenesis-related diseases is the activation of VEGF-C/VEGFR3 pathway [35, 36]. Although therapies targeting lymphangiogenesis, similar to antiangiogenic drugs directed at VEGF, hold promise for treating these conditions, they have not completely fulfilled expectations. Thus, it is required to elucidate the mechanism underlying lymphangiogenesis and explore alternative treatment strategies.

As a novel class of non-coding RNAs, tsRNAs have emerged as critical regulators of various cellular processes and have gained recognition for their involvement in a broad spectrum of physiological and pathological conditions [37, 38]. For example, tsRNA-04002 alleviates intervertebral disc degeneration by targeting PRKCA to inhibit apoptosis of nucleus pulposus cells [39]. tRF-Gln-CTG-026 mitigates liver injury by reducing global protein synthesis. Angiogenin-mediated tsRNAs regulate inflammation and metabolic disorders via targeting NLRP3 inflammasome [40]. We have revealed that tRNA-Cys-5-0007 plays a dual role of anti-angiogenesis and anti-inflammatory in ocular vascular disease [20]. This study focused on the functional characterization of tsRNA in lymphangiogenesis. We revealed a consistent down-regulation of tsRNA-0032 expression in both suture-induced murine corneal model and LPS-stimulated HLEC model. Gain- and loss-of-function experiments showed that tsRNA-0032 overexpression inhibits lymphangiogenesis, suggesting that tsRNA-0032 plays a suppressive role in this process.

Lymphangiogenesis, the growth of new lymphatic vessels, is a vital process driven by LECs. These specialized cells within the lymphatic system proliferate, migrate, and form tube-like structures in response to specific signals. LECs exhibit a unique metabolic profile, relying heavily on aerobic glycolysis to fuel their energy-demanding functions [12]. The Warburg effect is a metabolic phenomenon where cells prioritize glycolysis for energy production, even in oxygen-rich environments. Although less efficient than oxidative phosphorylation in terms of ATP yield, this shift is advantageous for rapidly dividing cells. By diverting glucose towards pyruvate through glycolysis, cells can effectively allocate resources for growth and development while minimizing oxidative stress caused by ROS [13]. When activated and transitioning from a resting state to a state of growth and movement, LECs significantly increase their glycolytic activity [41]. Impaired glycolysis severely affects cellular function. For example, deleting PKM2, a specific enzyme in podocytes, worsens angiotensin II-induced kidney damage, characterized by foot process effacement and proteinuria [42]. Similarly, glycolytic dysfunction in natural killer cells accelerates lung cancer progression [43]. PKM2 is the key enzyme in converting phosphoenolpyruvate to pyruvate, which is also essential for glycolysis [26]. Our study shows that tsRNA-0032 regulates glycolysis by targeting PKM2. Overexpressing tsRNA-0032 reduces glycolysis, leading to decreased production of pyruvate and lactate [44, 45]. These metabolic changes ultimately impair lymphatic endothelial cell function and suppress lymphatic vessel growth. Notably, abnormal levels of pyruvate and lactate have been linked to various diseases, including Alzheimer’s disease and tumor-draining lymph nodes [46, 47]. Pyruvate and lactate measurements demonstrate that increased tsRNA-0032 levels decreases pyruvate and lactate production, while decreased tsRNA-0032 levels increases their production. Therefore, tsRNA-0032 alters energy supply and the balance between metabolite production and consumption by targeting PKM2, thereby regulating lymphatic vessel growth.

To further clarify the clinical relevance of tsRNA-0032/PKM2 axis, we investigated the expression levels of tsRNA-0032 and PKM2 in corneal tissues obtained from keratitis patients post-corneal transplantation. Corneal samples from keratitis patients exhibits a significant reduction in tsRNA-0032 levels, accompanied by a marked increase in PKM2 expression compared to corneal tissues from healthy donors, offering compelling evidence of tsRNA-0032/PKM2 signaling pathway’s involvement in corneal lymphangiogenesis. The observed downregulation of tsRNA-0032 and upregulation of PKM2 in keratitis patients suggest a shift towards a more aggressive metabolic profile within lymphatic endothelial cells. This metabolic reprogramming may drive pathological lymphangiogenesis, which is often associated with chronic inflammation and tissue remodeling. Interventions aimed at restoring tsRNA-0032 levels or inhibiting PKM2 activity could offer novel strategies for mitigating lymphangiogenesis-related ocular diseases.

In conclusion, this study identifies tsRNA-0032 as a critical regulator of LEC function and lymphangiogenesis. We demonstrate that tsRNA-0032 significantly inhibits LEC proliferation, migration, tube formation, and sprouting in vitro. These inhibitory effects were further validated by in vivo model. Mechanistically, tsRNA-0032 was found to interact with Ago2 and target the glycolytic enzyme PKM2, thereby disrupting glycolytic reprogramming in LECs. These findings underscore the therapeutic potential of targeting tsRNA-0032/PKM2 axis in diseases involving pathological lymphangiogenesis.

## Supplementary information

Supplemental Material

Original Data

## Data Availability

All data supporting this study are presented in this published article and in its Supplementary information files.
