# Supplementary material for: Targeting glycolytic reprogramming by tsRNA-0032 for treating pathological lymphangiogenesis
Source: Cell Death Dis. 2025 Jan 28;16(1):51. doi: 10.1038/s41419-025-07366-w (PMC11772812; doi:10.1038/s41419-025-07366-w)
Supplement: Supplementary file 1 — Supplemental Material [file 41419_2025_7366_MOESM1_ESM.pdf]

Supplementary Information

Targeting glycolytic reprogramming by tsRNA-0032 for treating pathological lymphangiogenesis

Fan Ye [1, 2]*, Ziran Zhang [1]*, Lianjun Shi [1]*, Shuting Lu [1], Xiumiao Li [1],

Wan Mu [2, 3], Qin Jiang [1], Biao Yan [2]

[1] The Affiliated Eye Hospital, Nanjing Medical University, Nanjing 210000, China

[2] Department of Ophthalmology, Shanghai General Hospital, Shanghai Jiao Tong University School of Medicine, Shanghai 200080, China

[3] Eye Institute and Department of Ophthalmology, Eye & ENT Hospital, Fudan University, Shanghai 200031, China

* Fan Ye, Ziran Zhang, and Lianjun Shi contributed equally to this work.

Corresponding to:

Biao Yan E-mail: yanbiao@sjtu.edu.cn

Qin Jiang E-mail: jiangqin710@126.com

Supplementary Methods

Luciferase reporter assay

HLECs were seeded into 96-well plates and cultured in complete medium until reaching 60-70% confluence. They were co-transfected with either Luc-PKM2 WT, Luc-PKM2 Mut, or an empty vector, along with tsRNA-0032 mimics or negative control mimics using Lipofectamine 3000 (Invitrogen, USA, L3000015). After 24-h incubation, firefly and *Renilla* luciferase activities were detected by a luciferase reporter assay kit (Promega, USA, E1910).

Measurement of extracellular acidification rate (ECAR)

1×10^4 of HLECs were seeded onto Seahorse XFe96/XF Pro cell culture microplate (Agilent Technologies, 103794-100). They were cultured in XF base medium (pH 7.4) in a non-CO_2 incubator at 37°C for 1 h. Glucose (10 mM), glutamine (1 mM), 2-DG (50 mM), and oligomycin (1 μM) were sequentially added into the plates at specific time points following the manufacturer's protocol. ECAR was analyzed using the Seahorse XFe96 Analyzer (Agilent Technologies).

Quantification of pyruvate and lactate

1×10^6 of HLECs were seeded onto 6-well plate and received different treatments. Pyruvate and lactate levels were quantified using the pyruvate assay kit (Jiancheng Bioengineering Institute, China, A081–1-1) and lactate assay kit (Jiancheng Bioengineering Institute, China, A019-2-1). Absorbance was measured using the Multickan Skyhigh Microplate Reader (ThermoFisher Scientific, USA, A51119700DPC).

Supplemental figures

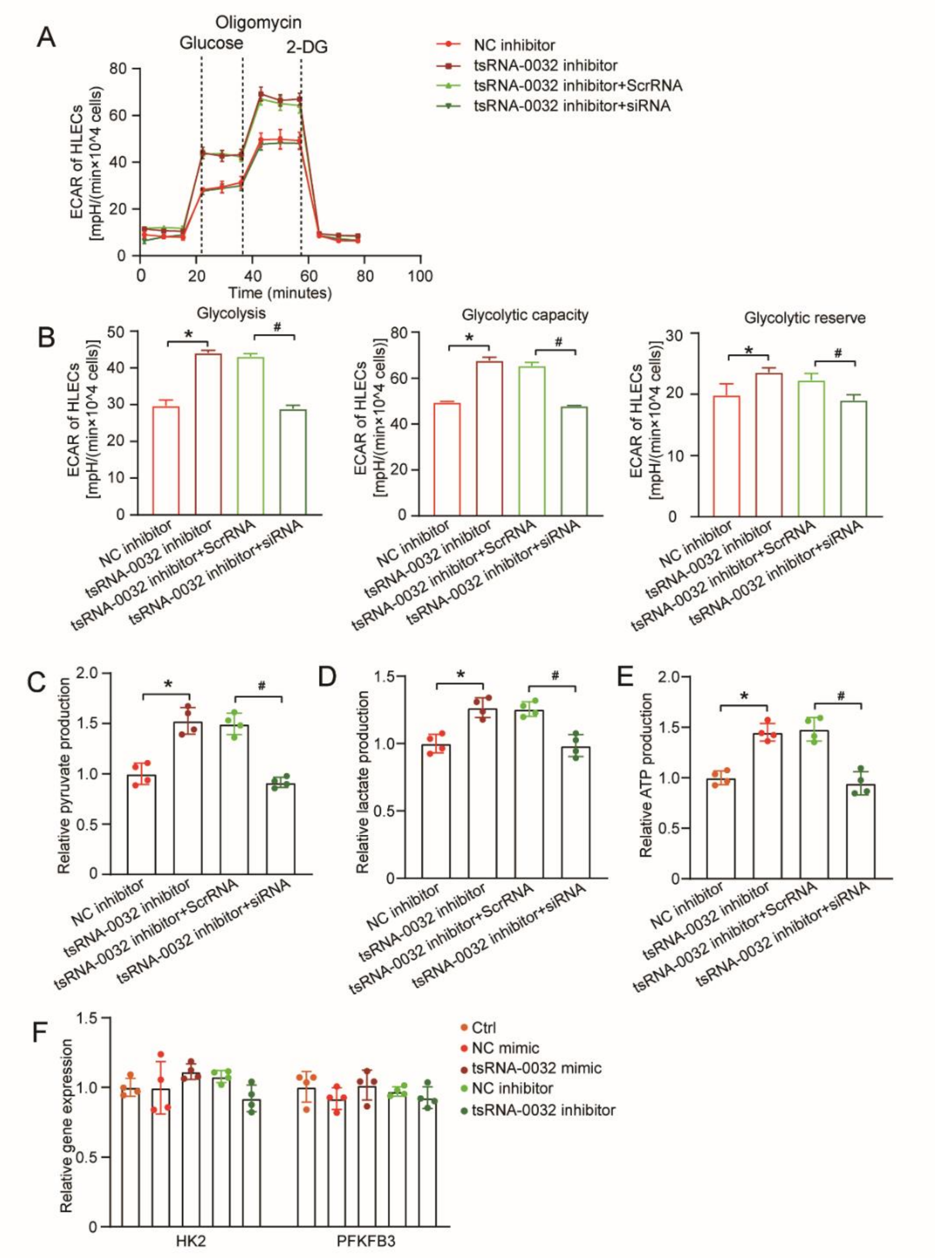

Figure S1. tsRNA-0032/PKM2 signaling axis alters glycolysis in HLECs

(A-E) HLECs transfected with negative control (NC) inhibitors, tsRNA-0032 inhibitors, tsRNA-0032 plus scramble siRNA (Scr siRNA), tsRNA-0032 plus PKM2 siRNA for 6 h. Seahorse XFe96 Analyzer was used to detect extracellular acidification rate (ECAR) of HLECs (A). Glycolysis, glycolytic capacity and glycolytic reserve were calculated from ECAR results (B). Pyruvate quantification kit, lactate quantification kit and ATP quantification kit were used to detect pyruvate, lactate and ATP production in HLECs (C-E, n = 4). One-way ANOVA followed by Bonferroni test; $^{*}P < 0.05$ between the marked groups. (F) HLECs were transfected with NC mimics, tsRNA-0032 mimics, NC inhibitors, tsRNA-0032 inhibitors, or were left untreated (Ctrl) for 6 h. The levels of HK2 and PFKFB3 RNA expression were determined by qRT-PCRs (n = 4, One-way ANOVA followed by Bonferroni test).

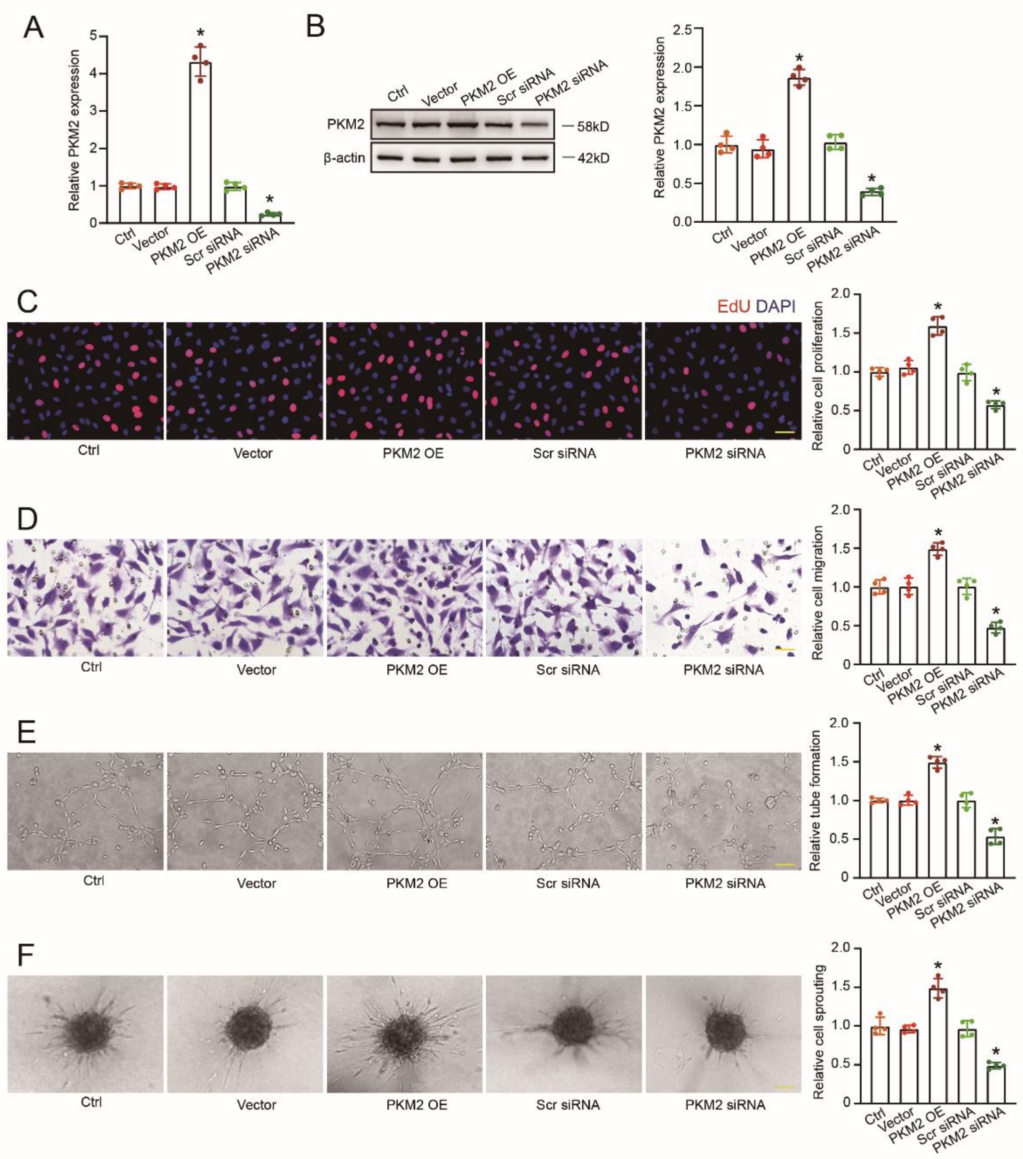

Figure S2. PKM2 regulates HLEC function *in vitro*

(A-F) HLECs were transfected with PKM2 null vector, PKM2 overexpression vector, scramble (Scr) siRNA, PKM2 siRNA, or were left untreated (Ctrl) for 6 h and then exposed to LPS (100 ng/mL) for 12 h. qRT-PCR assays and western blots were conducted to detect the expression of PKM2 in HLECs (A and B, n = 4, One-way ANOVA followed by Bonferroni test, $^{*}P < 0.05$ versus Ctrl group). Cell proliferation was detected by EdU staining. EdU, red; DAPI, blue. Scale bar, 20 μm (C, n = 4). Cell migration ability was detected by transwell assays. Scale bar, 20 μm (D, n = 4). Tube formation ability was detected by Martrigel assays. Scale bar, 100 μm (E, n = 4). Cell sprouting ability was detected by spheroid sprouting assays. Scale bar, 100 μm (F, n = 4). One-way ANOVA followed by post hoc Bonferroni test; $^{*}P < 0.05$ versus Ctrl group.

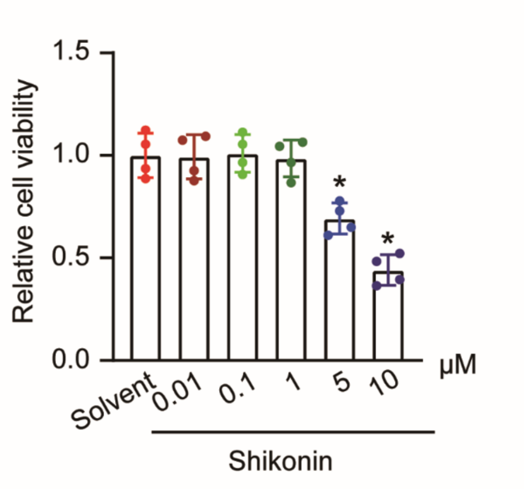

Figure S3. Effect of Shikonin treatment on the viability of HLECs

HLECs were pretreated with different concentrations of Shikonin for 6 h and then assessed for viability using CCK-8 assays (n = 4; One-way ANOVA followed by Bonferroni test, *$P < 0.05$).

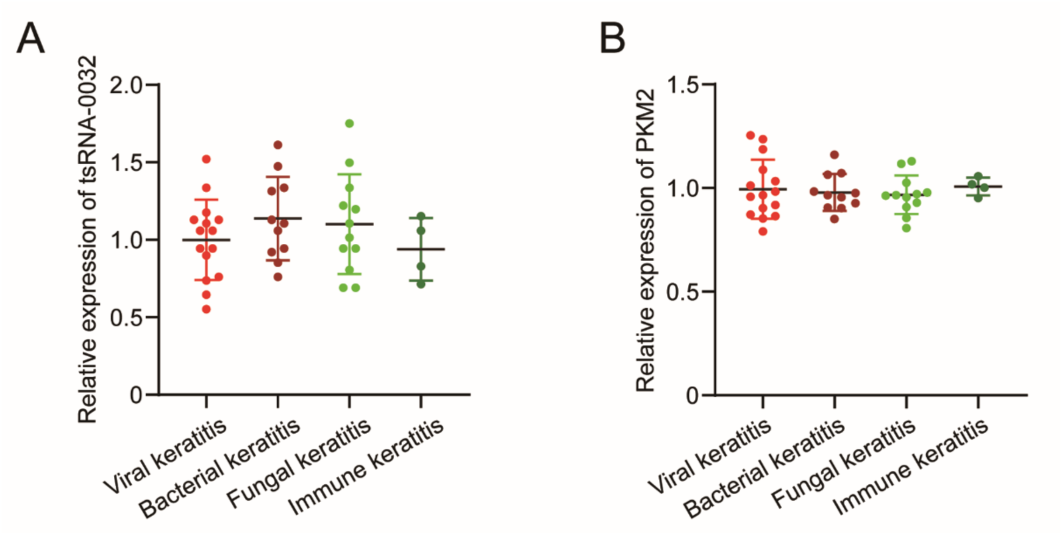

Figure S4. Detection of the expression difference of tsRNA-0032/PKM2 signaling axis among different types of keratitis

(A) qRT-PCR assays were conducted to detect the expression levels of tsRNA-0032 in various keratitis patients who received corneal allograft transplantation. (B) qRT-PCR assays were conducted to compare the expression levels of PKM2 in the same cohort of keratitis patients. One-way ANOVA followed by the Bonferroni post hoc test.

Table S1: Primers used in this study

Gene	Reverse primer (5'→3')	Reverse primer (5'→3')
ANG	AGGTGTCTTGCCCCTATGAC	ATCGTCTGTGATGGCTGTGC
Dicer	AGACTGTCGTGCCGTATTGG	TTCCTGTGCAGCTCCTCTTG
PKM2	ATGTCGAAGCCCCATAGTGAA	TGGGTGGTGAATCAATGTCCA
FASN	TGCGTGGCCTTTGAAATGTG	CTCCATGTCCGTGAACTGCT
HK2	CCTCCCAGCAAGTTTCACCT	ATTTTAGGACAGAGGCGGGC
PFKFB3	AATGCCCTCTTCACACCGTC	ACGGACTCCACGTTCAGGTA
AGO2	ACAGCCAGCATCGAACATGA	GAAATCTGGGACGGAAGGCA
β-actin	CTACCTCATGAAGATCCTCACCGA	TTCTCCTTAATGTCACGCACGATT

Table S2 Antibodies for western blot in this study

Anti-protein	Supplier	Catalog	Dilution
PKM2	Proteintech	15822-1-AP	1:1000
FASN	Proteintech	10624-2-AP	1:1000
AGO2	Abcam	ab186733	1:1000
β-actin	Proteintech	66009-1-Ig	1:1000
