# Supplementary material for: Targeting glycolytic reprogramming by tsRNA-0032 for treating pathological lymphangiogenesis
Source: Cell Death Dis. 2025 Jan 28;16(1):51. doi: 10.1038/s41419-025-07366-w (PMC11772812; doi:10.1038/s41419-025-07366-w)
Supplement: Supplementary file 2 — Original Data [file 41419_2025_7366_MOESM2_ESM.pdf]

Original western blots

Full unedited blots for Figure 4C

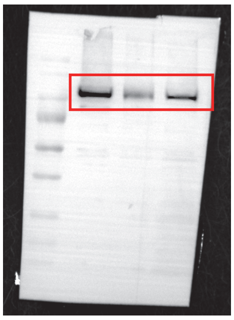

Ago2 97 kDa

Full unedited blots for Figure 4E

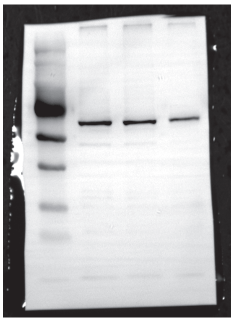

PKM2 58 kDa

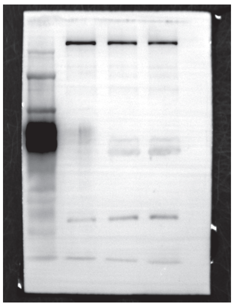

FASN 272 kDa

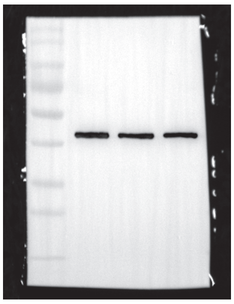

β-actin 42 kDa

Full unedited blots for Figure 4F

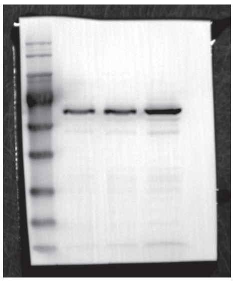

PKM2 58 kDa

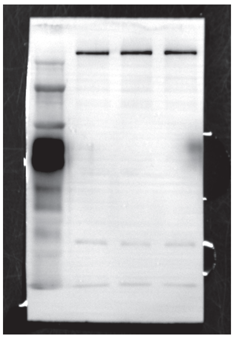

FASN 272 kDa

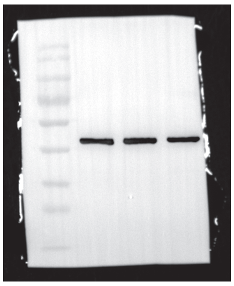

β-actin 42 kDa

Full unedited blots for Figure S3B

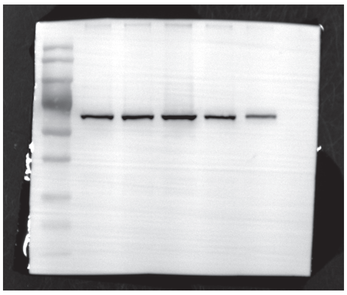

PKM2 58 kDa

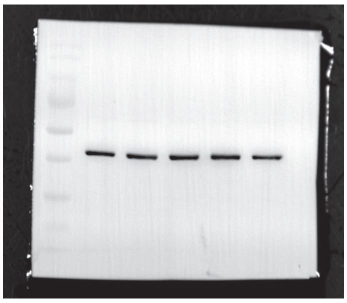

β-actin 42 kDa
